# Improving the Accuracy of Diagnosis for Multiple-System Atrophy Using Deep Learning-Based Method

**DOI:** 10.3390/biology11070951

**Published:** 2022-06-22

**Authors:** Yasuhiro Kanatani, Yoko Sato, Shota Nemoto, Manabu Ichikawa, Osamu Onodera

**Affiliations:** 1 Department of Clinical Pharmacology, Tokai University School of Medicine, 143 Shimokasuya, Isehara City 259-1193, Japan; 2 Division of Clinical Biostatistics, Shizuoka Graduate University of Public Health, 4-27-1 Kitaando Aoi-ku, Shizuoka City 420-0881, Japan; sato.yoko@s-sph.ac.jp; 3 Industrial & Digital Business Unit, Hitachi, Ltd., 1-5-2 Sotokanda, Chiyoda-ku, Tokyo 101-0021, Japan; shota.nemoto.nt@hitachi.com; 4 Department of Planning, Architecture and Environmental Systems, Shibaura Institute of Technology, 307 Fukasaku, Minuma-ku, Saitama City 337-8570, Japan; m-ichi@shibaura-it.ac.jp; 5 Department of Neurology, Brain Research Institute, Niigata University, 1-757 Asahimachidori, Chuo-ku, Niigata City 951-8585, Japan; onodera@bri.niigata-u.ac.jp

**Keywords:** multiple-system atrophy, artificial intelligence, pointwise linear model

## Abstract

**Simple Summary:**

Diagnosis of neurodegenerative diseases requires examination of a variety of characteristics. A definitive diagnosis is obtained using a comprehensive evaluation of family history, neurological findings, brain imaging, genetic testing, and other medical information. Multiple-system atrophy (MSA) is a neurodegenerative disease associated with autonomic dysfunction, parkinsonism, and cerebellar ataxia, and early diagnosis is difficult because the disease changes over time. The aims of this study were to examine whether machine learning can improve diagnostic accuracy using MSA case data from a national survey, and to identify the features that are important for differentiation among MSA subtypes using machine learning.

**Abstract:**

Multiple-system atrophy (MSA) is primarily an autonomic disorder with parkinsonism or cerebellar ataxia. Clinical diagnosis of MSA at an early stage is challenging because the symptoms change over the course of the disease. Recently, various artificial intelligence-based programs have been developed to improve the diagnostic accuracy of neurodegenerative diseases, but most are limited to the evaluation of diagnostic imaging. In this study, we examined the validity of diagnosis of MSA using a pointwise linear model (deep learning-based method). The goal of the study was to identify features associated with disease differentiation that were found to be important in deep learning. A total of 3377 registered MSA cases from FY2004 to FY2008 were used to train the model. The diagnostic probabilities of SND (striatonigral degeneration), SDS (Shy-Drager syndrome), and OPCA (olivopontocerebellar atrophy) were estimated to be 0.852 ± 0.107, 0.650 ± 0.235, and 0.858 ± 0.270, respectively. In the pointwise linear model used to identify and visualize features involved in individual subtypes, autonomic dysfunction was found to be a more prominent component of SDS compared to SND and OPCA. Similarly, respiratory failure was identified as a characteristic of SDS, dysphagia was identified as a characteristic of SND, and brain-stem atrophy was identified as a characteristic of OPCA.

## 1. Introduction

Multiple-system atrophy (MSA) is a neurodegenerative disorder characterized by progressive autonomic dysfunction, parkinsonism, and cerebellar and pyramidal features that occur in various combinations [1]. MSA used to be classified into olivopontocerebellar atrophy (OPCA), striatonigral degeneration (SND), and Shy-Drager syndrome (SDS); however, in the first diagnostic consensus on MSA, cases were classified as MSA-P for those with parkinsonism and MSA-C for those with cerebellar ataxia [2]. The term SDS, which had been used to describe MSA cases with prominent autonomic dysfunction, was formally taken out of use. In the second diagnostic consensus, MSA cases were categorized as definite, probable, and possible [3]. Since diagnosis of definite MSA requires pathological autopsy, early confirmation of a probable or possible case is clinically significant for patient management and disease-modifying therapy. In the second consensus, probable MSA was defined as a sporadic, progressive, and adult-onset (age ≥ 30 years) case with autonomic dysfunction and poorly L-DOPA-responsive parkinsonism (bradykinesia with rigidity, tremor, or postural instability) or cerebellar syndrome (gait ataxia with cerebellar dysarthria, limb ataxia, or cerebellar oculomotor dysfunction), while possible MSA was defined as a sporadic, progressive adult-onset case including parkinsonism or cerebellar ataxia and at least one feature suggesting autonomic dysfunction plus one other feature that may be a clinical or neuroimaging abnormality.

The accuracy of MSA diagnosis using the second consensus is 71% for probable MSA and 60% for possible MSA [4]. Therefore, an improvement in diagnostic accuracy is needed. Recently, artificial intelligence has been used to improve the diagnostic accuracy of neurodegenerative diseases, including MSA [5]. For example, differentiation of MSA from Parkinson’s disease was examined using brain imaging with computed tomography (CT) and magnetic resonance imaging (MRI) [6]. However, neurological findings and other medical information are important in MSA diagnosis [7], but few studies have examined the early diagnosis of neurodegenerative diseases by machine learning using datasets obtained in clinical practice as training data [8]. The issues associated with machine learning include standardization of the dataset [9] and evaluation of the obtained diagnostic probability, i.e., how to define the thresholds of certainty [10].

The second consensus diagnostic criteria for MSA do not cover all early-stage MSA cases [11]. SDS is not included as a subtype in this consensus but is known to be a clinical form of MSA with autonomic dysfunction as the primary symptom. The usefulness of the SDS concept in planning therapeutic trials is being evaluated, as early onset of autonomic dysfunction is a poor prognostic factor [12]. Therefore, we decided to use machine learning and conventional statistical methods to determine the features that influence diagnosis of the MSA subtypes in the earlier classification. The aim of the study was to evaluate the diagnostic accuracy of machine learning for the OPCA, SND, and SDS subtypes based on diagnoses by neurologists and to identify the important variables in classifying these subtypes.

Conventional statistical methods are mainly models based on linear or logistic regression between a small number of variables and outcomes. Machine learning can derive a broader range of standard variables using a neural network [13], but conventional machine learning is limited in visualization of this process [14]. Therefore, in this study, we used a deep learning-based method based on a pointwise linear model [15,16], which allows for correlations of each explanatory variable to express the target variable. We show that machine learning can increase the diagnostic accuracy of MSA to >80%, and we identify the important features for diagnosis of MSA and their relationship with each MSA subtype using the pointwise linear model. This method improves the diagnostic accuracy for early MSA and demonstrates the effectiveness of use of machine learning in validating diagnostic criteria.

## 2. Materials and Methods

### 2.1. Ethics

This study was performed under the ethical guidelines for medical and biological research involving human subjects issued by the Ministry of Education, Culture, Sports, Science, and Technology (MEXT), the Ministry of Health, Labor, and Welfare (MHLW), and the Ministry of Economy, Trade, and Industry (METI) in Japan. The ethics committee of the National Center of Neurology and Psychiatry approved the study (A2019-056; 14 January 2021). All patients gave written informed consent for registration in the Specified Disease Treatment Research Program. After submission of informed consent forms and approval of a review committee, including neurologists in their respective prefectural governments, personal information was anonymized, and cases were registered in the MHLW database [17]. The anonymized data were provided to us for analysis by the MHLW (9 March 2021).

### 2.2. Data and Diagnosis

Data were obtained from forms submitted to and digitized by the MHLW between FY2004 to FY2008. We first excluded duplicate cases and those without essential demographic data, such as onset age. Cases included in data analysis fulfilled the diagnostic criteria for MSA established by the MHLW Research Committee on MSA.

### 2.3. Items

The following demographic and clinical features were obtained from forms submitted as cases with MSA: sex, age, symptoms at onset, mode of onset, progression, neurological findings, autonomic findings, other neurological findings, brain images on CT and MRI, activities of daily living (ADL), and medication (Table 1), walking capacity, standing capacity/eyes open, finger-to-nose test, and knee–tibia test on the International Cooperative Ataxia Rating Scale (ICARS) [18], gait abnormalities due to parkinsonism on the Unified Parkinson Disease Rating Scale (UPDRS) [19], and bent posture, posture stability, tremor at rest, rigidity, finger taps, and rising from a chair on the Unified Multiple System Atrophy Rating Scale (UMSARS) [20]. Except for walking, ADLs were classified on a three-point scale (without assistance, with assistance, and unable). Walking was classified on a four-point scale (without assistance, with assistance, with a wheelchair, and unable) [21].

### 2.4. Classification of MSA Subtypes

A pointwise linear model, the deep learning-based method, was used for classification of MSA subtypes [22]. To evaluate over-learning in discriminant boundary generation, the training data were divided into a training and test dataset. Futhermore, to optimize the hyperparameters and evaluate prediction performance of the model, we conducted 10-fold double cross-validation using the training dataset, and the model with the best prediction accuracy (highest AUC) was adopted. The best hyperparameters are listed in Table 2. In this study, 60 items were used as explanatory valuables, and diagnosis was used as the objective variable (Table 1). Of the 3377 cases registered from FY2004 to FY2008, 3220 (851 SND, 359 SDS, and 2010 OPCA) were included, after exclusion of 157 cases with missing data. Ten cases of each MSA subtype were used for validation, and the remaining 3190 cases were used as the training dataset.

### 2.5. Extraction of Important Features Involved in Classification of MSA Subtypes

Diagnoses by neurologists were classified into three subtypes: SND, SDS, and OPCA. A pointwise linear model (implemented in Pytorch 1.5.1, Python 3.7.4) was used to identify important features in these diagnoses. Two datasets were used: dataset (a), which included the rank order of items; dataset (b), which did not include the rank order of items. Both datasets excluded medication information. The feature variables of the two datasets were classified into binary variables (B), categorical variables (C), ordinal variables (O), and quantitative variables (Q). All features included in datasets (a) and (b) are listed in Table 3. Binary variables were encoded as 1 or 0. One-hot encoding was used for categorical variables. Quantitative variables were normalized (mean = 0, standard deviation = 1), and ordinal variables were expressed on a scale of 1, 2, 3, etc., corresponding to the order of ranks. The final numbers of feature variables in datasets (a) and (b) were 58 and 126, respectively.

The predictive performance of the pointwise linear model was calculated using the area under the curve (AUC) evaluated by 10-fold double cross-validation (DCV). The one-vs.-rest strategy was used, in which a multiclass classification is split into one binary classification problem per class. Ultimately, the mean of the three different AUCs was considered to be the predictive performance.

To evaluate important features in each diagnosis, the importance score was defined using the weight vector. First, we calculated the sample-wise importance score $s_{k}^{\left(n\right)}$ for the *k*-th feature $x_{k}$ of a sample with index (n) as

$$s_{k}^{\left(n\right)}=\left|w_{k}^{\left(n\right)}x_{k}^{\left(n\right)}\right|,$$

where $w_{k}^{\left(n\right)}$ is the weight tailored for the *k*-th feature $x_{k}^{\left(n\right)}$ of sample (n) by the pointwise linear model. Next, for each subtype (e.g., SDS), the top 10% of features with sample-wise importance scores that were the largest in the classification model for each subtype were determined for each patient. Finally, the importance score was defined for each feature as the rate of samples whose top 10% features contained the feature.

### 2.6. Statistics

Descriptive statistics reported as counts (percentage) were used to describe the characteristics of the patients included in the analysis. A Kruskal–Wallis one-way analysis-of-variance-by-ranks test was performed to compare variables between MSA subtypes. All *p*-values are reported to three decimal places, with those less than 0.001 reported as *p* < 0.001. χ^2^ tests were used to compare categorical variables. Residual analysis was performed to determine which cell numbers in the cross-table represented sources of bias (*p* < 0.05) when significant bias was observed in a χ^2^ test (*p* < 0.05). All analyses were performed using STATA ver. 17.0 (Stata Corporation LLC, College Station, TX, USA).

## 3. Results

### 3.1. Patient Characteristics

A progressive course characterized the three MSA subtypes, and all had onset in the late 60s. Among early symptoms, ataxia (90.6%) was significantly more common in OPCA, parkinsonism (87.6%) was significantly more common in SND, and autonomic dysfunction (72.9%) was significantly more common in SDS. SND showed significantly more severe neurological findings than the other subtypes, and OPCA had a trend toward poorer finger-to-nose and knee–tibia tests and a higher frequency of dysarthria (79.3%). SDS showed significantly more autonomic dysfunction, especially respiratory failure (43.5%), than the other MSA subtypes. Brain CT and MRI revealed cerebellar atrophy common to all subtypes, with significant incidences of striatal atrophy/signal abnormality (58.7%) in SND and of brain-stem atrophy (79.3%) and a hot-cross-bun sign (47.9%) in OPCA. Regarding medication, dopamine receptor stimulants (40.5%) and amantadine hydrochloride (24.8%) were commonly used in SND, taltirelin hydrate (35.7%) was commonly used in OPCA, and droxidopa (37.7%) was commonly used in SDS (Table 4).

### 3.2. Diagnostic Probability Using the Point-Wise Linear Model

Every 10 cases were randomly selected from SND (851 cases), SDS (359 cases), and OPCA (2010 cases) diagnosed by neurologists in the deep learning-based method as the test dataset. The remaining cases were used as the training dataset. The AUC in the 10-fold DCV was 0.958 ± 0.001 in the training set and 0.959 ± 0.012 in the test dataset. The deep learning-based method resulted in high accuracies with a diagnostic probability of 0.852 ± 0.107 for SND and 0.858 ± 0.270 for OPCA. In contrast, SDS showed a significantly lower diagnostic probability of 0.650 ± 0.235 compared to SND and OPCA (*p* < 0.05). The diagnostic probability of SND, SDS, and OPCA for each case is shown in Table 5. Of the SDS cases classified as other subtypes, two were assigned to SND (cases 1 and 8) and one was assigned to OPCA (case 5); among OPCA cases, one was categorized as SND (case 2). On the other hand, in SND, all cases were classified into SND.

The deep learning-based method was used to analyze SND, SDS, and OPCA cases diagnosed by neurologists to determine the diagnostic probability by each subtype. Columns with the highest diagnostic probability were colored.

### 3.3. Identifying Important Features Using the Pointwise Linear Model

The pointwise linear model was used to extract important features that were closely associated with the diagnosis for each of the three MSA subtypes.

#### 3.3.1. Verification of the Prediction Performance for the Pointwise Linear Model

To investigate whether a specific rank in ordinal variables contributed to the classification of each diagnosis, we generated models with and without consideration of the rank order of items. Important features (score ≥ 0.3) were initially extracted from dataset (a) (number of feature variables: 58) or (b) (number of feature variables: 126) (Table 3). The AUCs for the models were calculated using 10-fold DCV. For each fold, a model was determined using the training set, and then the trained model was evaluated using the test set. The prediction performance for each learning model was evaluated as the mean AUC over the 10 folds. The AUCs for the training and test sets were 0.954 ± 0.001 and 0.956 ± 0.010, respectively, in the classification model including the order of neurological findings, and 0.962 ± 0.001 and 0.960 ± 0.010, respectively, in the model that did not include this order.

#### 3.3.2. Extraction of Important Features Closely Associated with the Diagnosis for the MSA Subtypes

Features with importance scores ≥ 0.30 were extracted from the pointwise linear model, i.e., features found to be important in ≥30% of patients. In the model including the order of severity, the finger-to-nose test ranked highest for all types, and ataxia onset was common to all types. Findings specific to each type included striatal atrophy/signal abnormality (score = 0.798) for SND, brain-stem atrophy (score = 0.347) for OPCA, and respiratory failure (score = 0.796) for SDS (Table 6a). If the order of severity was not taken into account (Table 6b), features common to all subtypes included autonomic dysfunction onset, parkinsonism onset, syncope, and striatal atrophy/signal abnormality. Common features were respiratory failure and head-up tilt test (positive) for SDS and OPCA, ataxia onset for SND and OPCA, and erectile dysfunction for SND and SDS. Dysphagia (score = 0.343) and walking capacity (normal) (score = 0.342) were specific to SND, severe constipation (score = 0.414) was specific to OPCA, and toileting (without assistance) (score = 0.433), urinary disturbance (score = 0.372), and urinary incontinence (score = 0.341) were specific to SDS.

To understand whether the occurrence of an event tended to raise the probability in an MSA subtype, the median regression coefficients (weights) tailored to each case are listed in Table 6, in addition to the score. Note that the presence/absence registration in the registry form is reversed from the usual relationship (presence = 1, absence = 0) because presence = 1, and absence = 2. Therefore, a negative value indicates a stronger correlation, while a positive value indicates a weaker correlation. On the other hand, for items classified as “O” in Table 3 (neurological findings and ADL), positive values indicate a positive correlation (severe disability). Therefore, finger-to-nose test, rigidity, and walking capacity are treated as “O” in Table 6a and “C” in Table 6b. In Table 6a, the finger-to-nose test showed positive values for all subtypes and negative values for ataxia onset. In Table 6b, autonomic dysfunction = −0.254 in SDS and ataxia onset = 0.366 in OPCA. For brain images from CT/MRI, the striatal atrophy/signal abnormality values were positive (0.111 and 0.130) in SDS and OPCA and negative (−0.161) in SND.

## 4. Discussion

MSA is a complex disease caused by a combination of parkinsonism, cerebellar ataxia, and autonomic dysfunction, and clinical presentation varies from onset of MSA [1]. The pathogenesis of MSA is thought to be accumulation of insoluble α-synuclein in neurons and oligodendroglia, which leads to progressive neurodegeneration [23]. In the latest international diagnostic consensus, diagnosis of MSA is classified into three stages: definite, probable, and possible. Since definite MSA requires an autopsy, a probable or possible clinical diagnosis is significant for disease management and selection of disease-modifying treatment [24]. However, in the current consensus, the probability of diagnosis is <70% [4]. A clinical trial targeting α-synuclein as a disease-modifying treatment for MSA is being planned, but since MSA is suspected only when clinical findings become apparent, early diagnosis is needed for development of treatment strategies [24].

Recent studies have examined utilization of artificial intelligence to improve the diagnostic accuracy of neurodegenerative diseases. However, these studies have mainly been limited to differential diagnosis using images and have not utilized medical information such as family history and neurological findings [6]. Moreover, it is challenging to make an early diagnosis by brain imaging alone, and a comprehensive evaluation of all medical information is necessary to improve diagnostic accuracy [25]. MSA is also a rare disease, and a nationwide collection of MSA cases requires the same diagnostic criteria, survey questions, and case validation framework to ensure data uniformity. For this reason, we used case information registered in a uniform nationwide survey. In addition, since newly registered cases were used, the data were likely to be from a relatively early point after MSA was suspected, as shown by the characteristics of the cases in Table 4.

Conventional statistical methods can reveal differences among subtypes, but machine learning has the advantage of linking all explanatory and objective variables for each case [26]. Thus, the diagnostic probability can be indicated by cases according to the MSA disease type with which it is strongly associated. The MSA cases used in this study were not classified as MSA-C or MSA-P, but as the subtypes of SND, SDS, and OPCA, and we machine-trained the data in these three subtypes to verify the diagnostic validity. Machine learning enables the diagnostic probability of each subtype to be shown for each case, which permits determination of this probability for each subtype. As shown in Table 5, some cases that were registered as SDS were classified as SND or OPCA. However, about 70% of the cases remained as SDS, which indicates that this subtype has features that differ from those of OPCA and SND. The diagnostic accuracies for SND and OPCA, which are strongly characterized by parkinsonism and cerebellar ataxia, respectively, exceeded 90%, suggesting that each of these populations has a homogeneous component.

One limitation of a machine learning is that the processing method is hidden, which limits the understanding of the relationship between objective and explanatory variables [14]. Therefore, we used a pointwise linear model, which can display the relationship between explanatory and objective variables as coefficients, to identify features involved in the diagnosis of each MSA subtype. This model was applied to the relationship between clinical information and diagnostic results for all items shown in Table 1. We note that the cases were registered at an early stage when MSA was suspected; hence, only the details of the medication could be ascertained, and results for drug responsiveness are still needed. We then tried to extract the important features that influence the classification of each subtype, excluding medication information. The accuracy of the machine learning model for MSA obtained using the pointwise linear model was not significantly affected by the amount of information used or the information structure, as the AUCs all showed high accuracy of ≥0.95, regardless of whether the severity of each item was ordered or not. Since the inclusion of a large amount of information can result in over-learning, which may lead to a decrease in model accuracy [27], we attempted to verify whether accuracy could be improved by narrowing down the items, but no significant differences were found. Therefore, the items ranked high for each condition in Table 6 were identified as important features for differentiation among the MSA subtypes.

**Figure 1:**
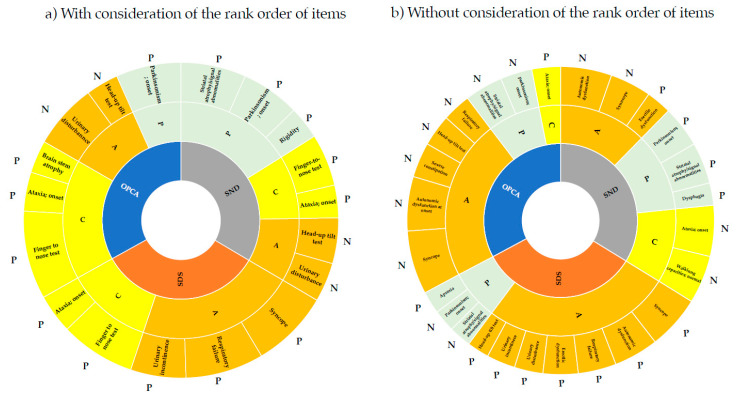
Features involved in diagnosis of MSA subtypes using machine learning. The items in Table 6a,b were divided into three components that were strongly associated with autonomic dysfunction (A), parkinsonism (P), and cerebellar ataxia (C). Analyses were performed with (**a**) and without (**b**) considering the order of severity of items for neurological findings and ADL. Based on the weight value, those with a strong relationship with the subtype were designated as P (positive) and those with a weak relationship with the subtype were designated as N (negative).

Results from the corresponding component analysis using the pointwise linear model showing the impact of autonomic dysfunction (A), parkinsonism (P), and cerebellar ataxia (C) components for each subtype are shown in Figure 1. When the order of severity was considered (Figure 1a), the A component was more important in SDS than in SND and OPCA, the P component (rigidity, parkinsonism onset, and striatal atrophy/signal abnormality) was more important in SND, and the C component (finger-to-nose test, brain-stem atrophy, and ataxia onset) was more important in OPCA. The finger-to-nose test and ataxia onset (C component) were commonly ranked high in all subtypes, reflecting the significantly greater impairment and more frequent occurrence of OPCA compared to the other two subtypes (Table 4).

The head-up tilt test (positive) and urinary disturbance high (A component) ranked high for SND and OPCA, but not for SDS, for which respiratory failure, syncope, and urinary incontinence ranked higher. These respective items tended to be significantly higher in SDS than in SND and OPCA, at 76.9%, 43.5%, and 57.8%, respectively (Table 4). In particular, respiratory failure, which is a poor prognostic factor in MSA, ranked high for SDS and may be a unique feature determining prognosis for this subtype [28]. For neurological findings and ADL, each item had more subitems according to the degree of disability. If order was not considered, the relationships among the 84 subitems included in these items and other items could be evaluated (Figure 1b). In contrast to Figure 1a, the P component commonly ranked high in all subtypes. In particular, parkinsonism onset and striatal atrophy/signal abnormality were common in all subtypes, while dysphagia was specific for SND and apraxia was specific for SDS. The higher frequency of cases assigned to this subtype compared to the other subtypes may be due to these specific findings. It is of note that dysphagia was identified as an important feature for SND because this condition is an additional feature of possible MSA-P and a poor prognostic factor for MSA-P [29].

Since the score indicates the importance of each item in the diagnosis, it is necessary to clarify whether this contributes positively or negatively to the diagnosis. Therefore, we examined the involvement of each item in the diagnosis in terms of positive and negative correlations using the median regression coefficient (weight) obtained in the pointwise linear model. With consideration of the rank order of the items, the finger-to-nose test tended to be positive, with a positive weight for all subtypes. In addition, ataxia onset was negative for all subtypes because presence/absence was reversed in the dataset structure (presence = 1, absence = 0) from the general dataset structure (presence = 1, absence = 2), indicating a tendency for all subtypes to have ataxia onset in common (Table 6a). Similarly, without considering the rank order of items, SDS tended to be associated with autonomic dysfunction and was less likely to be associated with parkinsonism (Table 6b). On the other hand, SDS showed a weight of −0.030 for apraxia, which indicates that apraxia is a significant differential feature in SDS. SND tended to be accompanied by erectile dysfunction as autonomic dysfunction and walking capacity normal, with a weight of −0.103. Given that the severity of walking capacity was included in the critical feature of the model with considering the order of severity of items, it suggests that “inability to walk normally” is more important than the severity of walking capacity in the diagnosis of SND. In OPCA, autonomic dysfunction and parkinsonism were less likely to be present.

This study shows that use of a machine learning can improve the diagnostic accuracy for MSA. However, important items in differential diagnosis need to be identified using a pointwise linear model, so that future studies of complex conditions such as MSA can be conducted. This will be a key aspect of development of diagnostic criteria for MSA.

## 5. Conclusions

In this study, we examined the feasibility of machine learning for differential diagnosis of MSA, which is characterized by a complex interplay of autonomic dysfunction, parkinsonism, and cerebellar ataxia that changes over time, and we identified important features in the diagnosis. Unlike conventional statistical methods that capture the characteristics of MSA subtypes, we were able to determine the influence of features that may have been overlooked in diagnosis by considering relationships among all the variables. Although poorly L-DOPA-responsive parkinsonism is a diagnostic criterion for MSA, treatment response could not be included in the machine learning because of the lack of data on response assessment after L-DOPA medication in this study. In this regard, it is not easy to objectively assess L-DOPA responsiveness at early diagnosis. On the other hand, it is possible to predict the prognosis from the information at the time of initial diagnosis by machine learning the long-term course of medical treatment.

## Figures and Tables

**Table 1:** Form for registration of cases of MSA.

Item	Option
Sex	1. Male, 2. female
Age	
Symptoms at onset	1. Ataxia
	2. Parkinsonism
	3. Autonomic dysfunction
Mode of onset	1. Mild, 2. subacute, 3. acute
Progression	1. Progressive, 2. arrested, 3. improved, 4. other
Neurological findings	1. Walking capacity
	2. Gait abnormalities due to parkinsonism
	3. Standing capacity, eyes open
	4. Bent posture
	5. Posture stability
	6. Finger-to-nose test
	7. Knee–tibia test
	8. Tremor at rest
	9. Rigidity
	10. Finger taps
	11. Rising from a chair
Autonomic findings	1. Head-up tilt test
	2. Syncope
	3. Urinary disturbances
	4. Urinary incontinence
	5. Erectile dysfunction (male only)
	6. Severe constipation
Other neurological findings	1. Dementia
	2. Hallucination (non-drug-induced)
	3. Aphasia
	4. Apraxia
	5. Agnosia
	6. Alien hand sign
	7. Vertical supranuclear gaze palsy
	8. Persistent Spontaneous Nystagmus
	9. Dysphasia
	10. Dysarthria
	11. Respiratory failure
	12.Tendon reflex
	13. Babinski reflex
	14. Other neurological findings
Brain images with CT/MRI	1. CT examination
	2. MRI examination
	3. Cerebellar atrophy
	4. Brain-stem atrophy
	5. Hot-cross-bun sign
	6. Striatal atrophy/signal abnormality
	7. Enlargement of 3rd ventricle
	8. Cerebral atrophy
	9. Cerebral white-matter lesion
ADL	1. Eating
	2. Bathing
	3. Hygiene
	4. Dressing
	5. Toileting
	6. Walking (more than 50 m)
	7. Climbing stairs
Medication	1. Taltirelin hydrate
	2. Protirelin tartrate hydrate
	3. Levodopa
	4. Dopamine receptor agonists
	5. Amantadine hydrochloride
	6. Anticholinergics
	7. MAO-B inhibitors
	8. Droxidopa
Diagnosis	1. SND 2. SDS 3. OPCA

**Table 2:** The best hyperparameters of the pointwise linear model for classification of MSA subtypes.

Hyperparameter	Best Parameter
Number of epochs	100
Number of inner layers	16
Size of layers	180
Label smoothing	0.055
Learning rate	1.83 × 10^−4^
Momentum	0.968
Optimization	adam
Dropout rate of inner layers	0.027
Dropout rate of input layer	0.171
Regularization coefficient	9.29 × 10^−5^
Ratio of L1 regularization	0.048

**Table 3:** Datasets used in the pointwise linear model.

		(a) With Rank Order of Items		(b) Without Rank Order of Items	
Item	Options for Item	Category #	Number of Feature Variables = 58	Category #	Number of Feature Variables = 126
Sex		B	1	B	1
Age		Q	1	Q	1
Symptoms at onset	1–3	B	3	B	3
Mode of onset		O	1	C	3
Progression		C	4	C	4
Neurological findings	1–11	O	11	C	62
Autonomic findings	1–6	B	6	B	6
Other neurological findings	1–11, 13	B	12	B	12
	12	C	3	C	3
Brain images with CT/MRI	1–9	B	9	B	9
ADL	1–7	O	7	C	22
Medication	1–8	n/a	0	n/a	0

# B, C, O, and Q indicate binary variables, categorical variables, ordinal variables, and quantitative variables, respectively.

**Table 4:** Characteristics of MSA according to clinical subtype.

Item	Category	SND	SDS	OPCA	*p*–Value
		894	377	2106	
Sex		1.21	0.41	0.92	<0.001
Age		67.4 ± 9.7	66.8 ± 10.6	64.8 ± 9.3	<0.001
Symptoms at onset, *n* (%)					
Ataxia	Yes	65 (7.3)	61 (16.2)	1907 (90.6)	<0.001
Parkinsonism	Yes	783 (87.6)	61 (16.2)	162 (7.7)	<0.001
Autonomic dysfunction	Yes	57 (6.4)	275 (72.9)	86 (4.1)	<0.001
Mode of onset, *n* (%)	Mild	835 (93.4)	333 (88.3) *	2017 (95.8) †	<0.001
	Subacute	56 (6.3)	39 (10.3)	84 (4.0) *	
	Acute	3 (0.3)	5 (1.3) †	5 (0.2)	
Progression, *n* (%)	Progressive	886 (99.1)	369 (97.9)	2087 (99.1)	0.137
	Arrested	8 (0.9)	5 (1.3)	11 (0.5)	
	Improved	0	2 (0.5)	4 (0.2)	
	Other	0	1 (0.2)	4 (0.2)	
Neurological findings	Scale				
1. Walking capacity	1–9	5.7 ± 2.5	5.0 ± 2.7	4.8 ± 2.3	<0.001
2. Gait abnormalities due to parkinsonism	1–5	3.3 ± 1.1	2.4 ± 1.4	1.9 ± 1.3	<0.001
3. Standing capacity, eyes open	1–8	4.6 ± 2.3	4.2 ± 2.4	4.2 ± 2.0	<0.001
4. Bent posture	1–5	2.6 ± 1.0	1.8 ± 0.9	1.6 ± 0.9	<0.001
5. Posture stability	1–5	3.4 ± 1.2	2.7 ± 1.5	2.5 ± 1.5	<0.001
6. Finger-to-nose test	1–5	2.1 ± 1.1	2.1 ± 0.9	2.6 ± 0.8	<0.001
7. Knee–tibia test	1–5	2.2 ± 1.2	2.2 ± 1.1	2.9 ± 1.0	<0.001
8. Tremor at rest	1–5	1.7 ± 0.9	1.4 ± 0.7	1.3 ± 0.6	<0.001
9. Rigidity	1–5	3.0 ± 0.8	2.0 ± 0.9	1.7 ± 0.9	<0.001
10. Finger taps	1–5	2.9 ± 0.9	2.1 ± 0.9	2.0 ± 1.0	<0.001
11. Rising from a chair	1–5	3.5 ± 1.3	2.8 ± 1.5	2.9 ± 1.5	<0.001
Autonomic findings, *n* (%)					
1. Head-up tilt test	Positive	348 (38.9)	310 (82.2)	818 (38.8)	<0.001
2. Syncope	Yes	166 (18.6)	290 (76.9)	269 (12.8)	<0.001
3. Urinary disturbances	Yes	465 (52.0)	300 (79.6)	839 (39.8)	<0.001
4. Urinary incontinence	Yes	335 (21.4)	218 (57.8)	517 (24.6)	<0.001
5. Erectile dysfunction (males only)	Yes	191 (47.1)	185 (69.2)	366 (33.4)	<0.001
6. Severe constipation	Yes	534 (59.7)	259 (68.7)	740 (35.1)	<0.001
Other neurological findings					
1. Dementia	Yes	127 (14.2)	52 (13.8)	212 (10.1)	<0.001
2. Hallucination (non-drug-induced)	Yes	37 (4.1)	10 (2.7)	24 (1.1)	<0.001
3. Aphasia	Yes	9 (1.0)	4 (1.1)	18 (0.9)	0.878
4. Apraxia	Yes	16 (1.8)	7 (1.9)	17 (0.8)	0.033
5. Agnosia	Yes	9 (1.0)	6 (1.6)	16 (0.8)	0.279
6. Alien hand sign	Yes	1 (0.1)	2 (0.5)	3 (0.1)	0.228
7. Vertical supranuclear gaze palsy	Yes	66 (7.4)	11 (2.9)	69 (3.3)	<0.001
8. Persistent spontaneous nystagmus	Yes	69 (7.7)	24 (6.4)	367 (17.4)	<0.001
9. Dysphasia	Yes	318 (35.6)	76 (20.2)	489 (23.2)	<0.001
10. Dysarthria	Yes	524 (58.6)	194 (51.5)	1670 (79.3)	<0.001
11. Respiratory failure	Yes	175 (19.6)	164 (43.5)	331 (15.7)	<0.001
12. Tendon reflex	Increased	363 (40.6)	133 (35.3) *	962 (45.7) †	<0.001
	Decreased	81 (9.1)	50 (13.3) †	177 (8.4)	
	Normal	450 (50.3)	194 (51.5)	967 (45.9) *	
13. Babinski reflex	Yes	189 (21.1)	78 (20.7)	391 (18.6)	0.489
Brain images with CT/MRI, *n* (%)					
1. Cerebellar atrophy	Yes	367 (41.1)	218 (57.8)	1980 (94.0)	<0.001
2. Brain-stem atrophy	Yes	323 (36.1)	173 (45.9)	1670 (79.3)	<0.001
3. Hot-cross-bun sign	Yes	189 (21.1)	81 (21.5)	1008 (47.9)	<0.001
4. Striatal atrophy/signal abnormality	Yes	525 (58.7)	44 (11.7)	111 (5.3)	<0.001
5. Enlargement of 3rd ventricle	Yes	64 (7.2)	29 (7.7)	140 (6.7)	0.674
6. Cerebral atrophy	Yes	116 (13.0)	52 (13.8)	148 (7.0)	<0.001
7. Cerebral white-matter lesion	Yes	47 (5.3)	24 (6.4)	70 (3.3)	<0.001
ADL	Scale				
1. Eating	1–3	1.5 ± 0.7	1.4 ± 0.6	1.3 ± 0.5	<0.001
2. Bathing	1–3	2.0 ± 0.7	1.8 ± 0.8	1.6 ± 0.7	<0.001
3. Hygiene	1–3	1.8 ± 0.7	1.6 ± 0.7	1.4 ± 0.6	<0.001
4. Dressing	1–3	1.8 ± 0.7	1.6 ± 0.7	1.4 ± 0.7	<0.001
5. Toileting	1–3	1.7 ± 0.7	1.6 ± 0.7	1.4 ± 0.2	<0.001
6. Walking (more than 50 m)	1–4	2.3 ± 1.0	2.1 ± 1.0	1.9 ± 1.0	<0.001
7. Climbing stairs	1–3	2.3 ± 0.8	2.1 ± 0.8	2.0 ± 0.8	<0.001
Medication, *n* (%)					
1. Taltirelin hydrate	1. Not used	786 (87.9) †	316 (83.8) †	1323 (62.8) *	<0.001
	2. Used	75 (8.4) *	48 (12.7) *	751 (35.7) †	
	3. Unknown	33 (3.7) †	13 (3.5)	32 (1.5) *	
2. Protirelin tartrate hydrate	1. Not used	824 (92.2) †	347 (92.0) †	1806 (85.8) *	<0.001
	2. Used	25 (2.8) *	23 (6.1) *	230 (10.9) †	
	3. Unknown	45 (5.0) †	17 (4.5) †	70 (3.3) *	
3. Levodopa	1. Not used	113 (12.6) *	257 (68.2) †	1752 (83.2) †	<0.001
	2. Used	775 (86.7) †	109 (28.9) *	288 (13.7) *	
	3. Unknown	6 (0.7) *	11 (2.9)	66 (3.1) †	
4. Dopamine receptor agonists	1. Not used	496 (55.5) *	318 (84.3)	1950 (92.6) †	<0.001
	2. Used	362 (40.5) †	42 (11.1) *	86 (4.1) *	
	3. Unknown	36 (4.0)	17 (4.5) †	70 (3.3)	
5. Amantadine hydrochloride	1. Not used	634 (70.9) *	335 (88.9)	1935 (91.9) †	<0.001
	2. Used	222 (24.8) †	26 (6.9) *	93 (4.4) *	
	3. Unknown	38 (4.3)	16 (4.2)	78 (3.7)	
6. Anticholinergic	1. Not used	775 (86.7) *	341 (90.5)	2000 (95.0) †	<0.001
	2. Used	79 (8.8) †	17 (4.5)	26 (1.2) *	
	3. Unknown	40 (4.5)	19 (5.0)	80 (3.8)	
7. MAO-B inhibitors	1. Not used	747 (83.6) *	347 (92.0)	2000 (95.0) †	<0.001
	2. Used	108 (12.1) †	11 (2.9)	26 (1.2) *	
	3. Unknown	39 (4.4)	19 (5.0)	80 (3.8)	
8. Droxidopa	1. Not used	693 (77.5) *	223 (59.2) *	1958 (93.0) †	<0.001
	2. Used	161 (18.0) †	142 (37.7) †	66 (3.1) *	
	3. Unknown	40 (4.5) †	12 (3.2)	82 (3.9)	

* Significantly smaller (*p* < 0.05) in residual analysis. † Significantly larger (*p* < 0.05) in residual analysis.

**Table 5:** Diagnostic probability estimated using the point-wise linear model.

	SND (*n* = 10)				SDS (*n* = 10)				OPCA (*n* = 10)		
	Diagnostic Probability				Diagnostic Probability				Diagnostic Probability		
No.	SND	SDS	OPCA	No.	SND	SDS	OPCA	No.	SND	SDS	OPCA
1	0.956	0.025	0.019	1	0.373	0.489	0.138	1	0.037	0.040	0.923
2	0.934	0.039	0.027	2	0.019	0.947	0.034	2	0.910	0.041	0.049
3	0.884	0.034	0.082	3	0.050	0.885	0.065	3	0.016	0.032	0.952
4	0.909	0.060	0.031	4	0.281	0.400	0.319	4	0.017	0.032	0.952
5	0.572	0.076	0.352	5	0.028	0.474	0.497	5	0.021	0.029	0.950
6	0.948	0.028	0.025	6	0.104	0.667	0.229	6	0.021	0.040	0.939
7	0.812	0.019	0.169	7	0.091	0.717	0.192	7	0.017	0.010	0.973
8	0.804	0.178	0.018	8	0.589	0.214	0.197	8	0.021	0.021	0.959
9	0.820	0.050	0.130	9	0.044	0.776	0.180	9	0.037	0.031	0.932
10	0.876	0.091	0.032	10	0.036	0.932	0.032	10	0.025	0.022	0.953

**Table 6:** Features involved in diagnosis of MSA using the pointwise linear model.

(a) With Consideration of the Rank Order of Items									
Rank	SND			SDS			OPCA		
	Feature	Score	Weight	Feature	Score	Weight	Feature	Score	Weight
1	Striatal atrophy/signal abnormalities	0.798	−0.143	Respiratory failure	0.796	−0.122	Finger-to-nose test	0.910	0.060
2	Parkinsonism onset	0.705	−0.288	Syncope	0.788	−0.139	Parkinsonism onset	0.694	−0.089
3	Finger-to-nose test	0.621	0.028	Finger-to-nose test	0.771	0.035	Urinary disturbance	0.517	0.104
4	Head-up tilt test	0.544	0.101	Urinary incontinence	0.562	−0.074	Ataxia onset	0.395	−0.458
5	Urinary disturbance	0.462	0.080	Ataxia onset	0.395	−0.222	Head-up tilt test	0.350	0.067
6	Ataxia onset	0.395	−0.183				Brain-stem atrophy	0.347	−0.155
7	Rigidity	0.378	0.023						
**(b) Without Consideration of the Rank Order of Items**									
**Rank**	**SND**			**SDS**			**OPCA**		
	**Feature**	**Score**	**Weight**	**Feature**	**Score**	**Weight**	**Feature**	**Score**	**Weight**
1	Autonomic dysfunction onset	0.720	0.022	Autonomic dysfunction onset	0.865	−0.254	Striatal atrophy/signal abnormalities	0.770	0.130
2	Syncope	0.639	0.173	Striatal atrophy/signal abnormalities	0.749	0.111	Syncope	0.720	0.155
3	Parkinsonism onset	0.628	−0.125	Parkinsonism onset	0.704	0.214	Parkinsonism onset	0.644	0.204
4	Striatal atrophy/signal abnormalities	0.603	−0.161	Respiratory failure	0.576	−0.084	Autonomic dysfunction onset	0.606	0.071
5	Erectile dysfunction	0.378	−0.020	Head-up tilt test	0.510	−0.133	Severe constipation	0.414	0.064
6	Ataxia onset	0.375	0.173	Syncope	0.495	−0.125	Ataxia onset	0.387	−0.366
7	Dysphagia	0.343	−0.025	Erectile dysfunction	0.444	−0.056	Head-up tilt test	0.376	0.061
8	Walking capacities, normal	0.342	−0.103	Toileting, without assistance	0.433	−0.081	Respiratory failure	0.342	0.047
9				Urinary incontinence	0.372	−0.053			
10				Apraxia	0.368	−0.030			
11				Urinary disturbance	0.341	−0.063			

## Data Availability

Not applicable.
